# Building Strong Foundations: Nonrandomized Interventional Study of a Novel, Digitally Delivered Fall Prevention Program for Older Adults

**DOI:** 10.2196/68957

**Published:** 2025-02-26

**Authors:** David Wing, Jeanne F Nichols, Hava Shoshana Barkai, Olivia Culbert, Daniel Moreno, Michael Higgins, Anna O'Brien, Mariana Perez, Hope Davey, Ryan Moran

**Affiliations:** 1 Herbert Wertheim School of Public Health and Human Longevity University of California, San Diego San Diego, CA United States; 2 Exercise and Physical Activity Resource Center University of California, San Diego San Diego, CA United States; 3 School of Medicine University of California, San Diego San Diego, CA United States

**Keywords:** exercise, older adults, digital intervention, Zoom, balance, posture, strength, fall prevention

## Abstract

**Background:**

Injuries from falls are a major concern among older adults. Targeted exercise has been shown to improve fall risk, and recommendations for identifying and referring older adults for exercise-based interventions exist. However, even when very inexpensive or free, many do not use available fall prevention programs, citing barriers related to convenience and safety. These issues are even greater among older adults residing in rural areas where facilities are less abundant. These realities highlight the need for different approaches to reducing falls in novel ways that increase reach and are safe and effective. Web-based delivery of exercise interventions offers some exciting and enticing prospects.

**Objective:**

Our objective was to assess the efficacy of the Strong Foundations exercise program to change markers of physical function, posture, balance, strength, and fall risk.

**Methods:**

Strong Foundations is a once weekly (60 minutes), 12-week iterative program with 3 core components: postural alignment and control, balance and mobility, and muscular strength and power. We used a quasi-experimental design to determine changes in physical function specific to balance, postural control, and muscular strength among older adults at low or moderate risk of falling.

**Results:**

A total of 55 low-risk and 37 moderate-risk participants were recruited. Participants significantly improved on the 30-second Chair Stand (mean change of 1, SD 3.3 repetitions; *P*=.006) and Timed Up and Go (mean change of 0.2, SD 0.7 seconds; *P*=.004), with the moderate-risk group generally improving to a greater degree than the low-risk group. Additionally, Short Physical Performance Battery performance improved significantly in the moderate-risk category (*P*=.02). The majority of postural measures showed statistically significant improvement for both groups (*P*<.05). Measures of “relaxed” posture showed improvements between 6% and 27%. When an “as tall as possible” posture was adopted, improvements were ~36%.

**Conclusions:**

In this 12-week, iterative, web-based program, we found older adults experienced improvement not only in measures used in clinical contexts, such as the 30-second Chair Stand and Timed Up and Go, but also contextualized gains by providing deeper phenotypical measurement related to posture, strength, and balance. Further, many of the physical improvements were attenuated by baseline fall risk level, with those with the highest level of risk having the greater gains, and, thus, the most benefit from such interventions.

## Introduction

The absolute number of adults older than 65 years of age has been steadily growing for decades and now outnumbers the number of children younger than 18 years of age [1]. In addition, estimates indicate that older adults will represent greater than 23% of the population in the United States by 2035 [1]. Injuries resulting from falls are the largest cause of accidental death and mobility-related disability among older adults. This is likely because nearly one-quarter of community-residing adults older than 65 years of age fall annually, and that number rises to almost half of those older than 80 years of age [2]. Particularly concerning is the fact that once an individual starts to fall, there are often additional falls. Indeed, evidence from numerous sources suggests that between 10% and 44% of older adult patients who have fallen will sustain additional falls in the following year [3-7].

Targeted strength and balance exercises have consistently been shown to improve fall risk, and accordingly, recommendations for identifying and referring older adults for exercise-based interventions have been developed. Public health and clinically oriented authorities, including the Centers for Disease Control and Prevention (CDC) and the US Preventive Services Task Force, have acknowledged the importance of risk assessment and physical activity (PA) in improving fall risk for older adults. The CDC’s Stopping Elderly Accidents and Deaths Initiative (STEADI) has also established an evidenced-based questionnaire that assesses known risk factors to help clinicians stratify individuals by risk category to identify those who should be best served by fall prevention programs [8-10].

Many exercise programs for older adults exist. While some health insurance may include access to programs such as Silver Sneakers and EnhanceFitness PA at no additional charge [11], many older adults may have to pay substantial fees to participate. Further, even when very inexpensive or free, many eligible older adults do not use these programs, citing several barriers [11,12]. These include personal preferences (dislike of gymnasiums and lack of knowledge regarding appropriate activity), environmental factors (difficulty in reaching gymnasiums or classes, concerns about bad weather and driving, concerns about being “old” in a gymnasium environment), and structural factors (limited number of facilities and concerns about instructor expertise with older adults) [12]. These issues are even greater among older adults residing in rural areas. Rural residents have fewer facilities for guided PA, more limited transportation infrastructure [13], and less exercise expertise than their urban counterparts [14]. Confounding these difficulties, ongoing concerns exist surrounding the COVID-19 transmissibility alongside the availability, and desirability, of in-person programs. Since the height of the pandemic, there has been a growth in available options for in-person exercise programs. However, while vaccines and exposure have decreased the likelihood of serious infection, there remains some risk associated with (large) in-person gatherings, particularly among the more vulnerable older adult population.

These realities highlight the need for different approaches in addressing the public health challenge of reducing the likelihood of falls in older adults in novel ways that increase reach and are safe and effective. Digital delivery of exercise interventions offers some exciting and enticing prospects, as not only can this platform eliminate barriers related to difficulty with transportation and concerns about the gymnasium environment, but it can also bring highly qualified experts to individuals who are the most in need. These include those experiencing specific conditions that require special instruction and those living in rural and geographically remote areas. Given that older adults are increasingly confident and competent using the internet and videoconferencing tools [15,16], developing interventions that leverage this technology to improve access to fall-risk reduction exercise programs, including balance, posture, and strength training, is both worthwhile and feasible.

Despite these possibilities, there remains limited research on digital group–based exercise programs, particularly those focused on fall prevention. Further, the available evidence is generally drawn from relatively small samples [17-19], or the studies have been conducted in populations with specific medical conditions or comorbidities such as diabetes [20,21] or mild cognitive impairment [22]. To the authors’ knowledge, there are only 2 randomized studies with a sample size greater than 20 that have focused on healthy older adults. Although the exercise modalities differed, following the intervention, participants did have generally positive findings regarding improvements in strength and balance [23,24]. However, neither intervention included exercises specific to postural control, a factor strongly hypothesized to influence fall risk [25,26]. Moreover, instruction during exercise sessions in nearly all studies we reviewed, regardless of the health status of the participants, was generalized to the entire class due to the difficulty of the instructor speaking to any 1 person to correct technique without interrupting the flow of the class.

Therefore, we developed and digitally deployed an evidence-based exercise program, Strong Foundations (SF), with instruction provided in real time, to enhance physical function and ultimately prevent falls among older adults. The aim of this study was to evaluate the SF program’s effectiveness in improving the posture, balance, and strength of older adults using valid, reliable, and widely used measures of physical function.

## Methods

### Ethical Considerations

This study was approved by the institutional review board (#806696) at the University of California, San Diego, and informed consent was gathered from all participants. They were further informed that they did not have to complete any measurements and were free to withdraw from the exercise sessions at any time. Participants were additionally provided with US $20 for each laboratory visit to cover travel costs and were given resistance bands to be used during the exercise intervention (value ~US $10). All data were de-identified prior to analyses with a code to participant numbers and identities held only by the PI and study coordinator and maintained in protected file servers. 

### Study Design and Recruitment

We used a quasi-experimental design to determine the efficacy of digitally delivered exercise for improving physical function specific to balance, postural control, and muscular strength among older adults at low or moderate risk of falling. Several strategies were used to recruit participants: (1) posters displayed at older adult living facilities and community centers; (2) emails sent to existing lists of older adults who previously expressed interest in research studies in general, and in fracture prevention, specifically; and (3) announcements made following educational presentations on fall and fracture prevention delivered digitally or in person to various groups of older adults in the community. Those who expressed interest were contacted by study personnel and screened by phone for eligibility. Inclusion criteria included being 60 years and older, having an internet-enabled device with a screen of 7+ inches (tablet, laptop, or similar), and scoring less than 8 on the CDC’s STEADI questionnaire. Participants were further stratified based on their answers to the STEADI questionnaire into low- and moderate-risk groups with a maximal score of 3 and 7, respectively [27].

### Intervention Design

Designed by 2 exercise physiologists working in consultation with both a medical doctor and a doctor of physical therapy, the SF program is a once weekly (60 minutes), 12-week iterative program with 3 core components: postural alignment and control, balance and mobility, and muscular strength and power. All instructors leading the training had, at minimum, a bachelor’s degree in an associated field (kinesiology, exercise physiology, etc) and licensing as a personal trainer from an accredited national institution (American College of Sports Medicine, American Council on Exercise, and National Academy of Sports Medicine). In addition to the weekly group class, participants received both printed and recorded instruction regarding how to safely complete the exercises without supervision and were “assigned” homework materials and encouraged to practice 2 or more additional times per week preferably with a family member or friend present for safety.

All of the exercises introduced throughout the course were designed to be appropriate for an older adult population and were standardized so that participants received the same basic instruction, but the level of difficulty was scaled (and coached) based on individual capability, experience, and musculoskeletal limitations. Each week, new foundational exercises identified as being important to maintaining strength, postural control, balance, and mobility, and also relevant to specific daily activities (eg, picking up an object from the floor to place it on a countertop) were added to the class. In addition, over time, most exercises initially taught as isolated movements were expanded to more complicated, multijoint and multiplanar compound movements, once again with the intent to mimic daily movement.

### Home Setup and Safety Concerns

One to two weeks before the first exercise class, participants were emailed instructions for setting up their exercise space at their residence. This included having, at minimum, a 6×6 ft uncluttered exercise space on either a nonslippery floor or an area with wall-to-wall carpet (no loose rugs) and adequate open space surrounding the designated exercise space. In addition, if possible, they were encouraged to have a small amount of wall space immediately behind their exercise area to allow for intermittent wall exercises. Participants were also provided with a set of resistance bands with the level of difficulty determined based on their self-described strength and were instructed to ensure that they had a chair without wheels (and preferably without arms) available for chair-based exercises and to assist with balance during standing exercise.

### Intervention Delivery

One of the novel features of this program is the delivery of semi-individualized instruction in real time within a small group setting. To this end, classes were designed to have 10-15 participants. A single lead instructor provided verbal instruction while demonstrating each exercise. Simultaneously, at least 1, and sometimes 2, additional instructors provided individualized instruction to participants who were performing an exercise incorrectly or those who were ready to move on to a more difficult progression, with a particular emphasis on exercise form and safety. This allowed all participants to develop competency with key exercises while allowing those with experience and confidence to progress to more advanced movements in a timely manner.

In-person classes were offered once per week and lasted slightly longer than 1 hour. Classes began with approximately 5 minutes of aerobic warm-up during, which participants were encouraged to move somewhat vigorously in order to raise their heart rate and elicit blood flow to the working muscles. Following the warm-up, each class had different foci as shown in Table 1. Regardless of the focus of the week, 5-10 minutes were spent teaching the week’s foundational exercises with particular emphasis on explaining proper form. In the early weeks of the intervention, participants were instructed to complete the exercises slowly, paying particular attention to ensure that they were feeling the target muscles activate or engage in the appropriate anatomical area. Individualized feedback from an assistant instructor correcting errors in technique was emphasized during this time, making sure that each participant received feedback from an instructor at least once during the class. As the weeks progressed, the lead instructor cued participants to increase the resistance or increase the speed, or increase both the speed and resistance concurrently during strength training exercises, as tolerated, and also increase balance challenges if they felt safe doing so.

**Table 1:** Weekly foci and foundational exercises.

Week	Foundational exercise 1	Foundational exercise 2	Foundational exercise 3	Foundational exercise 4	Focus of the week
1	Neutral spine or marionette pose	Pelvic tucks or tilts	Hip hikes	Hip hinge	Posture
2	Quick feet drills	Review hip hinge	“Traditional abs”	Self-test: static balance	Balance
3	Front and lateral arm raises (with band)	Hip hinge (review) with squat or chair sit or stand	Reverse lunge	Heel toe raises	Strength
4	Neutral spine and pelvic tuck or tilt—standing	Static balance	Reverse lunge with progression	Heel toe raises with tennis ball	Combine 3 pillars
5	Sit and stand transitions	Around the clock steps	Hip hinge with choice of W/T/Y/I arm extensions	Modified or regular jumping jacks	Floor to stand transition—floor exercises
6	Hip hinge (review) with squat or chair sit or stand	Lunge with chair progression	Single leg heel raises	Hing hinge+picking up objects	Compound strength movements
7	Drinking bird	Squat to lateral leg raise	Single leg heel raises	Toe raise walk around chair	Increase balance challenge
8	Warrior 2 with chair sit	Wall sit with wood chop	Introduce pivot	Quick feet multidirectional	Odd impact or multidirectional movements
9	Weight transfer with split stance	Squat into a high knee and arm reach	Pivot	Head rotations in a tandem stance	Combine balance with compound movements
10	Single leg stance with band pull downs and serial 7’s	Lunge with chair support and head rotation	Standing superman with cognitive challenge	Goal post with arm slides	Introduce cognitive challenge
11	Reaching squats	Lateral jacks	Vertical push-ups	Knee drivers	Speed or power movements
12	N/A^a^	N/A	N/A	N/A	Collaborative workout

^a^N/A: not applicable.

For the remainder of the class, a variety of exercises focused on strength, balance, and posture (alone and in combination) in line with the focus of the week were completed in sets of 3 to 5 with pauses between sets to cue the next set of exercises. During the final 5 minutes, participants were guided through a brief cool down that incorporated stretching of the muscles used (most) during the exercise session.

Participants were also provided a secure link to a public-facing web-based video library that hosted a recording of the exercise session and a written description of the foundational exercises emphasized each week. Participants were further encouraged to complete 2 additional exercise sessions weekly. They also were provided a “mid-week challenge” 3 days after the weekly class session. These challenges included video presentations on the importance of posture in preventing falls, selected yoga-based movements for balance, and other short (5-10 minutes) presentations designed to keep participants engaged with the larger fall prevention focus of SF. Finally, participants were reminded of the importance of cardiovascular exercise and encouraged to walk or do other aerobic activities regularly.

### Laboratory Measures

#### Overview

All measures were completed at baseline, prior to the first exercise class. Follow-up visits were completed anytime from the day after the 11th class through 2 weeks following the completion of the last (12th) exercise session. Participants completed their physical measures in the following order:

#### Balance and Physical Function

In-laboratory testing of balance and physical function included the Short Physical Performance Battery (SPPB) [28] combined with computerized dynamic posturography to quantify postural sway and the Timed Up and Go (TUG) [29].

Participants completed the standard SPPB, which includes (1) a standing balance challenge, in which the participant is asked to stand as still as possible with an increasingly narrow base of support; (2) regular walking speed at a “regular” pace on a 4-m course; and (3) leg strength gathered from the time taken to complete 5 chair stands. Continuous scores were normalized with a maximal score of 12 (4 points per measurement category) [28]. In an effort to address possible ceiling effects associated with the balance component of the SPPB, the BTrackS Balance Plate (version 7.5; Balance Tracking Systems) was used to capture overall postural sway, measured in centimeters.

Participants completed the TUG at both a normal walking speed and as fast as possible. Participants began in a seated position and were instructed to rise without using their arms to push off, walk 3 m, negotiate around an obstacle (rubber cone), return to their chair, and sit back down. Participants were allowed 1 practice trial and then completed each test twice with the best time (ie, lowest) score used for statistical analysis.

#### Postural Assessments

Posture was measured using a variety of methods designed to identify areas of postural deficiency. Specifically, for general posture, standing height was measured in two ways using a stadiometer (SECA 213) while participants were instructed to (1) “stand as you normally do” and (2) “stand as tall as possible.” Both measurements were taken at the top of the breath cycle. To identify cervical and upper thoracic postural decline, the occiput to wall distance (OWD) [30] was measured in the same 2 manners (ie, regular and tall stance). More specifically, OWD was measured by having participants stand against a wall with their heels and buttocks touching the wall or as close as they could get comfortably. They were then instructed to look straight ahead (neutral head position) and hold still while the distance from the occiput to the wall was measured in centimeters. Finally, the Debrunner kyphometer and flexiruler were used to measure thoracic and lumbar curvature using previously published methodology [31,32]. Briefly, the Debrunner kyphometer provided an angle of curvature of the thoracic spine when placed in the joint space between T2 and T3 on one end and T12 and L1 on the other while participants stood in a normal or relaxed manner. The flexiruler is moldable and is placed with one end at the base of C7 and the other in the joint space between L5 and S1. The molded ruler is then traced onto a piece of paper, from which the kyphotic index was calculated by 2 independent raters as a function of the thoracic length and thoracic width using standardized procedures previously described (width/length×100) [31]. The average of the 2 raters was used for analyses.

#### Functional Muscular Strength

All participants completed a 30-second Chair Stand (30CS) and grip strength test. A subset (initial 3 cohorts of recruited participants) also completed isometric strength testing of hamstrings or quadriceps and trunk or lumbar muscle groups (procedure described in Isometric Muscular Strength section).

The 30CS used a chair with a seat height of 17 inches. Participants were instructed to keep their feet flat on the floor with their arms folded across their chest and touching their chest throughout the test. To be counted, the participant had to rise to a fully upright position and then return to a seated position while maintaining a fairly vertical body position (avoiding excessive forward lean). If participants did not maintain proper form, they were coached, but time was not stopped.

#### Hand Grip Strength

Hand grip strength was measured using an adjustable grip strength dynamometer (Jamar Plus Digital Hand Dynamometer). During the baseline visit, the grip or handlebar of the dynamometer was adjusted so the second joint of the fingers fits around the handle with handle size (1-5) recorded and used during subsequent visits. Participants were familiarized with the measurement by performing one submaximal effort on each hand. After becoming comfortable with the procedure, participants were instructed to hold the dynamometer with their arms at their side. They were then coached to take a deep breath in and squeeze as hard as possible as they exhaled. Measurement staff provided encouragement throughout each attempt. The measurement was repeated twice on each hand, alternating between the dominant and nondominant hand, with the highest score for each hand recorded in kilograms.

#### Isometric Muscular Strength

Isometric strength of the hamstrings, quadriceps, and spinal extensor muscles was measured using the Biodex System 4 PRO dynamometer (version 4.60; Biodex Medical Systems). Specifically, isometric strength of the hamstring and quadriceps muscle groups was measured with the leg held in at 45, 75, and 90 degrees of knee flexion. Participants performed 3 repetitions of 5-second maximal isometric contraction, extension followed by flexion, with 5 seconds of rest between repetitions at each angle. A 2-minute rest period was given between sets or leg positions. To decrease participant burden, only 1 leg was tested with participants indicating if either of their legs or knees had any previous injuries, or any current pain, after which the “healthiest” underwent testing. The average maximal contraction at each angle was recorded, and scores were combined into a flexion and extension composite score.

The Biodex Dual Position Back Ex/Flex Attachment, seated-compressed variation (isolated lumbar position), was used to measure the isometric strength of the trunk at 0 degrees of spinal flexion-extension (seated up-right). One set of 3 repetitions of 5-second maximal isometric contractions for both trunk flexion and extension was completed by participants. A 10-second rest was given between repetitions. To minimize the risk of injury, participants were instructed to slowly and gradually increase their muscle engagement to a volitional maximum and not attempt any sudden or explosive movement against the resistance.

#### Aerobic Fitness

Participants were asked to walk continuously for 2.5 minutes at their normal walking speed on a 50-m corridor [33]. During the walk, participants wore a chest strap–based heart rate monitor (Polar H10). Total distance, average heart rate, and peak heart rate were recorded.

### Statistical Analyses

All statistical analyses were conducted using SPSS (version 27; IBM Corp). As this was largely designed as a feasibility study to determine the acceptability of this novel intervention delivery method and the degree to which the intervention could improve metrics of strength, balance, and posture, power calculations were not conducted. Instead, the sample size was primarily determined by safety concerns involving class size, which we settled on 10-15 per class after conducting 3 brief, 4-week pilot sessions beginning with ~6 participants, increasing to 8-12 to keep class size manageable. The sample size was also restricted by fiscal constraints.

Descriptive statistics (percentages, means, and SDs) were used to characterize demographic variables and identify potential outliers. Change scores were derived by subtracting baseline values from follow-up values on an individual level.

Independent 2-tailed *t* tests were conducted to assess differences across groups at baseline. Paired sample *t* tests were conducted to evaluate the effects of the intervention on the measures of interest, with analyses conducted both for the total population and also with participants divided into fall-risk groups, which were assessed independently of each other. Associations between changes in measures of strength, balance, and posture and number of classes attended were examined using Pearson correlation without controlling for any covariates. When associations were observed, univariate linear modeling was conducted, with sex, chronological age, and baseline score included as covariates. These were included based on the known systematic declines in performance in older individuals, the possibility of systematic differences between male and female individuals, and the differences in potential change associated with baseline performance.

## Results

### Overview

**Figure 1:**
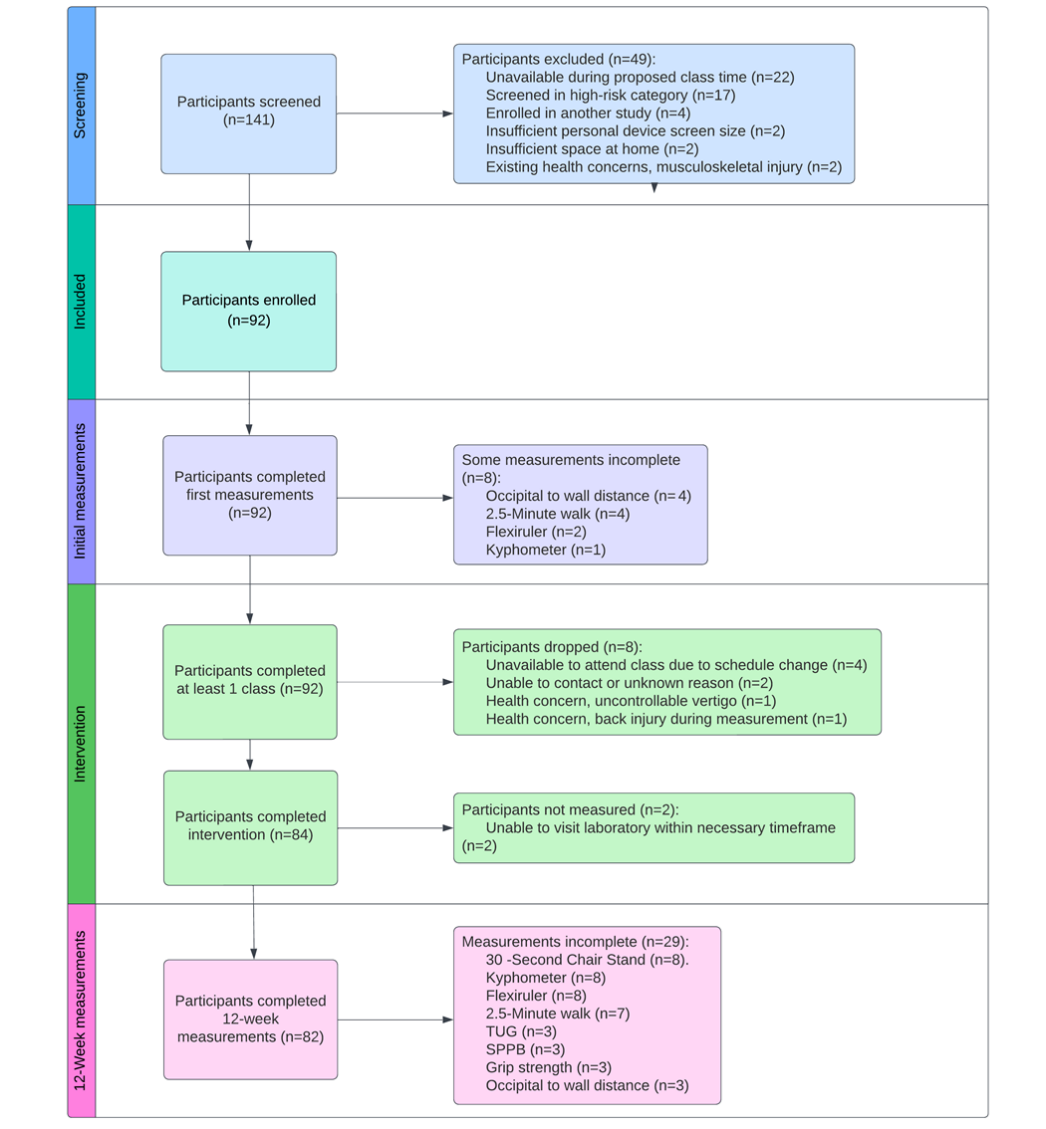
Participant recruitment flow. The sum of the breakdown of excluded participants at the initial and 12-week measurements exceeds the total number of excluded participants, as some individuals missed more than one assessment. SPPB: Short Physical Performance Battery; TUG: Timed Up and Go.

The number of participants screened, enrolled, and followed through the final measurement is shown in Figure 1.

### Population Descriptive Data

A total of 92 participants were enrolled across 8 cohorts with 55 low-risk and 37 moderate-risk individuals assigned to their own risk-group classes (n=4 classes per risk group). Key demographics and population descriptive data are shown in Table 2. The majority of the population was female (n=77, 84%) and White (n=74, 80%). Participants were largely well educated, with all participants having some college degree, and 91% (n=84) having a bachelor’s degree or beyond. Additionally, although very few were currently employed (n=15, 16%), the majority (n=55, 60%) of participants were at a high socioeconomic status (>US $100,000 income), with 19 (21%) individuals earning more than US $200,000 per year.

As expected, based upon the group stratification, there was a difference in STEADI fall risk score between groups. Additionally, those at moderate risk (STEADI >3 but <8) were slightly heavier (low risk: mean 64.2, SD 11.3 kg and medium risk: mean 71.6, SD 17.7 kg; *P*=.008) and with a higher BMI (low risk: mean 23.8, SD 3.9 kg/m^2^ and medium risk: mean 26.5, SD 5.2 kg/m^2^; *P*=.003).

Performance metrics at baseline and following the 12-week intervention are shown in Table 3. There was a difference at baseline between the low- and moderate-risk groups on the 30CS (*P*<.001), TUG (*P*=.002), walking distance at normal speed over 2.5 minutes (*P*<.001), SPPB (*P*<.001), and the OWD measures of posture (normal stance: *P*=.007 and tall stance: *P*=.005). In all of these cases, the moderate-risk group had worse performance compared to the low-risk group. However, strength and balance and some measures of posture (flexiruler and Debrunner kyphometer) were not different by risk category.

**Table 2:** Demographic data of participants by fall-risk group.

		Total (N=92)	Low risk (n=55)	Moderate risk (n=37)	*P* value	
Female, n (%)		77 (84)	47 (85)	30 (81)	.58	
**Race, n (%)**						N/A^a^
	Asian	8 (9)	5 (9)	3 (8)		
	Black	2 (2)	1 (2)	1 (3)		
	Hispanic	3 (3)	1 (2)	2 (5)		
	White	74 (80)	45 (82)	29 (78)		
	Other	5 (5)	3 (5)	2 (5)		
Age (years), mean (SD)		72.0 (6.6)	71.3 (6.9)	73.0 (6.2)	.25	
Height (cm), mean (SD)		163.9 (7.7)	164.0 (6.0)	163.7 (9.9)	.43	
Weight (kg), mean (SD)		67.1 (14.6)	64.2 (11.3)	71.6 (17.7)	*.008* ^b^	
BMI (kg/cm^2^), mean (SD)		24.9 (4.6)	23.8 (3.9)	26.5 (5.1)	*.003*	
**Income (US $ per year), n (%)**						
	<50,000	12 (13)	4 (7)	8 (22)	.11	
	>50,000 but <100,000	24 (26)	15 (27)	9 (24)	N/A	
	>100,000	55 (60)	35 (64)	20 (54)	N/A	
STEADI^c^ risk score (0-12 range), mean (SD)		3.3 (2.0)	2.0 (1.4)	5.2 (1.3)	*<.001*	
Classes attended, mean (SD)		9.7 (3.1)	9.4 (3.5)	10.3 (2.3)	.08	

^a^N/A: not applicable.

^b^Values in italics format indicate statistical significance at or below *P*<.05.

^c^STEADI: Stopping Elderly Accidents and Deaths Initiative.

**Table 3:** Intervention effects on performance-based measures by fall-risk category.

	Total (n=82 except where noted)				Low (n=48 except where noted)				Moderate (n=34 except where noted)		
	Preintervention, mean (SD)	Postintervention, mean (SD)	*P* value	Preintervention, mean (SD)		Postintervention, mean (SD)	*P* value	Preintervention, mean (SD)		Postintervention, mean (SD)	*P* value
Kyphotic index (%)	11.4 (3.4)	10.7 (3.5)	*.007* ^a^	11.4 (3.5)		10.8 (3.3)	.20	11.4 (3.3)		10.5 (3.8)	*.008*
Kyphotic angle (degrees)	40.3 (11.9)	37.7 (13.7)	*.005*	40.3 (10.9)		38 (11.2)	*.02*	40.2 (13.6)		37.3 (17.3)	*.008*
OWD^b^—normal stance (cm)	4.8 (3.8)	3.5 (3.8)	*<.001*	3.9 (3.4)		2.7 (3.2)	*.004*	6.1 (4)		4.7 (4.3)	*<.001*
OWD—tall stance (cm)	2.5 (3.0)	1.6 (2.7)	*<.001*	1.8 (2.7)		0.8 (2.1)	*.002*	3.6 (3.3)		2.8 (2.9)	*.01*
30-Second Chair Stand (repetitions)	12.9 (4.9)	14.3 (5.3)	*.006*	14.7 (4.4)		15.6 (5.1)	.44	10.1 (4.3)		12.3 (5.0)	*.002*
Grip combined (kg)	47.8 (13.8)	47.6 (14.4)	.54	47.9 (14.1)		47.1 (14.1)	.18	47.8 (13.7)		48.1 (15.0)	.60
Isometric leg strength extension (kg) (n=26 with/19 low and 7 moderate)	199 (80.6)	206 (79.1)	*.02*	196.1 (84.6)		193.7 (65.7)	.07	213.4 (62.1)		253.3 (112.6)	.11
Isometric leg strength flexion (kg) (n=26 with/19 low and 7 moderate)	93.9 (42.9)	98.7 (41.1)	.13	94.2 (45)		97.0 (37.7)	.29	92.3 (34.5)		105.2 (56.0)	.20
Isometric trunk strength extension (kg) (n=26 with/19 low and 7 moderate)	70.3 (28.1)	83.0 (40.0)	*.03*	72.5 (28.5)		90.9 (38.0)	*.01*	64.6 (28.1)		61.8 (40.6)	.75
Isometric trunk strength flexion (kg) (n=26 with/19 low and 7 moderate)	43.9 (21.1)	51.8 (20.4)	*<.001*	45.5 (19.8)		54.1 (17.9)	*.002*	39.7 (25.4)		45.9 (26.4)	.16
SPPB^c^ (scored from 0 to 12)	11 (1.4)	11.2 (1.2)	.13	11.5 (0.9)		11.5 (1.0)	.40	10.2 (1.6)		10.8 (1.4)	*.02*
Timed Up and Go (seconds)	6.9 (1.7)	6.7 (1.5)	*.004*	6.3 (1.2)		6.1 (1.2)	*.02*	7.9 (1.9)		7.5 (1.6)	.09
Postural sway (cm)	91.4 (31.1)	92.6 (35)	.43	88.4 (27)		86.8 (25.7)	.98	96.7 (37.2)		101.5 (44.8)	.16
Distance during 2.5-minute walk (m)	196.3 (30.7)	193.1 (31.6)	.18	209.2 (29.2)		202.5 (29.9)	.05	179.9 (24.5)		181.0 (33.4)	.75

^a^Values in italics format indicate statistically significant values with a *P* value of less than .05.

^b^OWD: occiput to wall distance.

^c^SPPB: Short Physical Performance Battery.

### Balance and Physical Function

On average, at baseline, participants were able to complete slightly more than the minimum number of chair stands (n<12) to be considered free from disability [34]. However, 38 (41%) individuals were below that level. In contrast, on average, participants were well above the minimum threshold to identify disability for the TUG and SPPB (>13.5 seconds and <9, combined score respectively) [35,36], with 0 for TUG and 14 (16%) for SPPB below the threshold. In terms of the effects of the intervention, there were no significant changes in postural sway during static balance stances associated with the SPPB. However, overall participants significantly improved on the 30CS and TUG (mean change of 1.4 repetitions: *P*=.006 and 0.2 seconds: *P*=.004, respectively). While not always statistically significant, the moderate-risk group improved to a greater degree on both of these assessments than the low-risk group (although both groups improved). Additionally, SPPB performance improved significantly in the moderate-risk category (*P*=.02).

### Posture

Although there were no inclusion or exclusion criteria related to posture, there were a meaningful number that displayed suboptimal posture. Indeed, depending upon the measurement tool and associated categorical cut-points used, between 7% and 26% of the total sample were hyperkyphotic (flexiruler: n=7, 8%; kyphometer: n=12, 13%; OWD [normal stance]: n=24, 26%). The majority of postural measures showed statistically significant improvement following the intervention. Indeed, measures of “relaxed” posture showed improvements between 6% (n=5; Debrunner kyphometer) and 27% (n=22; OWD). When an “as tall as possible” posture was adopted for the OWD, the improvements were greater at ~36% change from baseline. These changes are even more impressive when individuals who had no opportunity for improvement are removed (ie, individuals who scored 0 on OWD at baseline). Specifically, when the 18 individuals standing normally and 47 as tall as possible who scored 0 are removed, overall change scores with normal stance were 1.7 cm, and with tall stance were 2.2 cm.

### Muscular Strength

We did not observe significant differences in grip strength (*P*=.54) However, as mentioned earlier, 30CS is a marker both of functional movement but also muscular strength (and endurance) and had significant changes across the population (*P*=.006) Finally, in the subgroup that completed isometric testing, there was a significant improvement in knee extension (*P*=.02) and both trunk extension and flexion (*P*=.03 and <.001, respectively).

### Aerobic Fitness

There were no significant changes in measures of aerobic fitness measured by the 2.5-minute walk at normal speed.

### Associations Between Class Attendance and Functional Measures

When exploring associations between change scores and rates of attendance, the TUG (*r*=–0.255; *P*=.03) and 30CS (*r*=0.27; *P*=.02) were significantly correlated; however, there were no significant associations between class attendance and changes in physical function on the majority of other variables (*P*>.05). Regression analysis controlling for sex, age, baseline measurement, and number of classes attended indicated that attendance was not a significant predictor of change in TUG, although including it did slightly improve the model’s predictive value (from *R*=0.45 to 0.49). However, attendance was a significant predictor of improvement in the 30CS (unstandardized β=.502; *P*=.02). This indicates that for each class attended, there was a 0.5 increase in the change score in terms of number of repetitions. Model summary and individual contributions of variables and covariates are shown in Tables 4 and 5.

**Table 4:** Linear regression analysis of functional fitness and attendance change in the Timed Up and Go^a^.

Predictors	Unstandardized β (SE)	Standardized β coefficient	*t* test (*df*=72)	*P* value
Constant	–.382 (0.981)	N/A^b^	–0.389	.70
Sex^c^	–.066 (0.19)	–0.036	–0.346	.73
Chronological age (years)	.031 (0.012)	0.297	2.657	*.01^d^*
Baseline TUG^e^ (seconds)	–.174 (0.047)	–0.417	–3.72	*<.001*
Attendance	–.073 (0.04)	–0.191	–1.81	.07

^a^Model summary: *R*=0.485; *R*^2^=0.235; adjusted *R*^2^=0.193; standard error of the estimate=0.6118; *P*=.07.

^b^N/A: not applicable.

^c^Male: n=1 and female: n=2.

^d^Values in italics format indicate significance at, or below, the level of .05.

^e^TUG: Timed Up and Go.

**Table 5:** Linear regression analysis of functional fitness and attendance change in the 30-second Chair Stand^a^.

Predictors	Unstandardized β (SE)	Standardized β coefficient	*t* test (*df*=72)	*P* value
Constant	6.172 (5.689)	N/A^b^	1.085	.28
Sex^c^	–.785 (0.991)	–0.089	–0.792	.43
Chronological age (years)	–.096 (0.06)	–0.185	–1.6	.11
Baseline 30-second Chair Stand	–.156 (0.079)	–0.227	–1.978	.05
Attendance	.502 (0.21)	0.266	2.393	*.02^d^*

^a^Model summary: *R*=0.374; *R*^2^=0.14; adjusted *R*^2^=0.092; standard error of the estimate=3.173; *P*=.02.

^b^N/A: not applicable.

^c^Male: n=1 and female: n=2.

^d^Values in italics format indicate significance at, or below, the level of .05.

### Adverse Events

Based upon weekly tracking, there were several minor adverse events throughout the intervention period. However, none were related to the exercise intervention, and only 1 minor event was possibly related to the measurements. Additionally, there were no falls associated with the intervention, either while supervised during class or performed on nonclass days.

## Discussion

### Principal Findings

In this 12-week, iterative, web-based program, we found that older adults experienced an improvement in several measures widely used to evaluate fall risk and mobility-related disability [7,28,34]. While the magnitude of most of the changes was small, they were primarily consistent in the expected direction, which is not surprising because the program was designed to be largely instructional rather than a progressive training program. Instructors emphasized proper form and execution while maintaining good posture. Increases in the intensity of resistance exercises and challenges to balance and mobility exercises were only introduced once instructors observed consistent execution of movements. Importantly, no injuries were sustained during the remote delivery of our program or during the recommended home exercise practice.

Most muscular strength and physical function measures showed favorable changes in both the low- and moderate-risk groups, although not all results reached statistical significance. Nevertheless, the observed changes have clinical relevance. For instance, the intervention induced meaningful improvements in measures commonly used in clinical settings, such as the 30CS and TUG tests. Notably, many improvements varied by risk level, with greater gains seen in those at higher fall risk, who arguably had more to gain from the intervention. Specifically, participants in the moderate-risk group completed an additional 2 chair rises (a 21% improvement) by the end of the 12 weeks. Given that both leg strength and muscular endurance are essential for scoring well on the 30CS, the observed improvement in chair stands may be attributed to the focused practice of squat and related exercises in class as well as encouragement for similar home practice. While upper body or arm strength is important to overall good health and function, they do not substantially contribute to fall prevention and were therefore not heavily emphasized in our home practice recommendations. Similarly, the lack of significant changes in aerobic capacity was expected based on the program’s goals and the unique challenges of aerobic activities conducted via a Zoom format (Zoom Video Communications).

Additionally, these findings also contextualize participant gains by providing deeper phenotypical measurements related to posture, more sophisticated measures of strength, and balance. For example, there were positive changes recorded in measures of thoracic kyphosis and OWD across both risk groups. This is important because posture is often overlooked in many group exercise programs for both younger and older adults. In this study population, posture measured as the kyphotic angle using a Debrunner kyphometer decreased 2-3 degrees (*P*=.02 and .07 for low- and moderate-risk groups, respectively), while the OWD decreased between 1 and 1.4 cm (all *P*<.05) among participants in both groups over the once-weekly, 12-week exercise program. Considering that thoracic kyphosis typically worsens steadily over time beginning as early as age 40 years [37-39], observing a reversal of this condition within just 12 weeks of targeted exercise is highly encouraging. This is particularly meaningful, given the observed links between hyperkyphosis and both fall risk [26,40] and physical function [41,42].

Although there were no significant differences observed in grip strength, it is likely that the prescribed exercises contributed to an enhancement of the participant’s back strength [43] and chest flexibility, which helped alleviate the tightness in chronically shortened pectoral muscles [26]. These improvements not only reflected positively in participants’ attempts at maintaining “good” posture (as measured by OWD while standing tall) but also in their “normal” posture as assessed by various tools. These changes in both “good” and “normal” posture likely stemmed from the early introduction of postural control as a foundational element of the program as well as ongoing reminders throughout the course for participants to practice maintaining good posture during daily activities. It is worth noting that postural control is seldom included in group exercise training programs, making this approach particularly novel and engaging, thereby providing participants with more opportunities to learn and improve compared to more traditional exercise modalities such as aerobic activities or strength training.

While there have been some similar findings regarding the possibility of providing digital exercise instruction to older adults [17-19], this is, to our knowledge, the first study to robustly measure a large population of older adults across multiple metrics of physical health and function. Our findings clearly demonstrate the potential to deliver an effective fall prevention program through a technological interface. The program was well-received, as indicated by an attendance rate exceeding 80% (mean attendance 9.7 of 12), and no adverse events were reported in association with the exercise regimen. Additionally, the observed improvements in leg strength, postural control, and overall mobility, both independently and synergistically, show promise for reducing falls among older adults, including those at substantial risk.

### Limitations

Despite several strengths of this study, certain limitations must be acknowledged. Chief among them is the absence of a control group, which limits our ability to ascertain whether observed changes were influenced by external factors. A control group would also help clarify the significance of nonsignificant changes observed in some metrics, particularly in light of the expected performance declines over time. Additionally, although there were improvements in most metrics of strength and posture, balance as measured in this context was not improved. This may be a function of the method of measuring static balance (ie, postural sway during the SPPB). However, it may be that the program’s focus on balance was not sufficient to induce changes over 12 weeks. Furthermore, unlike many exercise interventions for adults of all ages, we did not emphasize cardiovascular health other than recommending daily general PA. While recognizing the importance of aerobic activity for overall health and longevity, instructional focus on this area was minimal; encouragement for at-home exercise emphasized practicing postural, strength, and balance-based exercises. Finally, the relatively affluent nature of the population limits the generalizability of both the results and the ability to disseminate this program to a wider population.

With the success of this program and the limitations noted earlier in mind, future research should explore the possibility of deploying this intervention in populations that are (1) of a lower overall socioeconomic status and (2) more remote from the location of intervention deployment. In addition, developing these materials into other languages in a culturally appropriate manner offers substantial potential to expand the reach to other populations who would benefit from the opportunity to receive at-home fall-risk training.

### Conclusions

The 12-week SF program improved physical function and posture in ways consistent with reduced fall risk. The real-time instruction also helps ensure program safety and adherence. As such, this program shows promise in digitally delivering needed public health interventions targeting fall risk among older adults.

## Abbreviations

30CS: 30-second Chair Stand
CDC: Centers for Disease Control and Prevention
OWD: occiput to wall distance
PA: physical activity
SF: Strong Foundations
SPPB: Short Physical Performance Battery
STEADI: Stopping Elderly Accidents and Deaths Initiative
TUG: Timed Up and Go

## Data Availability

The datasets used and analyzed during this study are available from the corresponding author on reasonable request.
